# Quality of the diet during the COVID-19 pandemic in 11 Latin-American countries

**DOI:** 10.1186/s41043-022-00316-8

**Published:** 2022-08-04

**Authors:** Samuel Durán-Agüero, Alfonsina Ortiz, Patricio Pérez-Armijo, María Fernanda Vinueza-Veloz, Israel Ríos-Castillo, Saby Camacho-Lopez, Brian M. Cavagnari, Edna J. Nava-González, Valeria Carpio-Arias, Karla Cordón-Arrivillaga, Saby Mauricio-Alza, Jhon Jairo Bejarano Roncancio, Beatríz Nuñez-Martínez, Gabriel González-Medina, Sonia Ivancovich, Eliana Romina Meza-Miranda, Leslie Landaeta-Díaz

**Affiliations:** 1 Escuela de Nutrición y Dietética, Facultad de Ciencias Para el Cuidado de la Salud, Universidad San Sebastián, Santiago de Chile, Chile; 2 Departamento de Nutrición, Facultad de Ciencias de La Salud, Universidad Católica del Uruguay, Montevideo, Uruguay; 3 Departamento de Medicina Preventiva y Salud Pública, Facultad de Farmacia, Universidad del País Vasco UPV/EHU, Barrio Sarriena, Spain; 4 PSICOMED Research Group, School of Medicine, Faculty of Public Health, Escuela Superior Politécnica de Chimborazo, Riobamba, Ecuador; 5 Neurocience Department, Erasmus MC, Rotterdam, The Netherlands; 6 Organización de las Naciones Unidas para la Alimentación y la Agricultura (FAO), Oficina Subregional de la FAO Para Mesoamérica, Panama City, Panama; 7 Escuela de Nutrición, Facultad de Medicina, Universidad de Panamá, Panama City, Panama; 8 Nutrir México, Mexico, Mexico; 9 Escuela de Nutrición, Facultad de Ciencias Médicas, Pontificia Universidad Católica Argentina, Puerto Madero, Argentina; 10 Facultad de Salud Pública y Nutrición, Universidad Autónoma de Nuevo León, San Nicolás, México; 11 Grupo de Investigación en Alimentación y Nutrición Humana (GIANH), Facultad de Salud Pública, Escuela Superior Politécnica de Chimborazo, Riobamba, Ecuador; 12 Unidad de Investigación en Seguridad Alimentaria y Nutricional (UNISAN), Escuela de Nutrición, Facultad de Ciencias Químicas y Farmacia, Universidad de San Carlos de Guatemala, Guatemala, Guatemala; 13 Universidad Privada Norbert Wiener, Lima, Peru; 14 Departamento de Nutrición Humana, Facultad de Medicina, Universidad Nacional de Colombia, Bogotá, Colombia; 15 Universidad Autónoma de Asunción, Asunción, Paraguay; 16 Departamento de Salud Pública, Facultad de Medicina, Pontificia Universidad Católica de Chile, Santiago, Chile; 17 Asociación Costarricense de Nutricionistas y Dietistas, San José, Costa Rica; 18 Centro Multidisciplinario de Investigaciones Tecnológicas, Universidad Nacional de Asunción, San Lorenzo, Paraguay; 19 Facultad de Salud y Ciencias Sociales, Universidad de Las Américas, Av. Walker Martínez 1360, Piso 3 Edificio A, La Florida, Santiago, Chile

**Keywords:** Food, COVID-19, Food quality, Quarantine, Education, Weight

## Abstract

**Background and objectives:**

The confinement by COVID-19 has affected the food chain and environments, which added to factors such as anxiety, frustration, fear and stress have modified the quality of the diet in the population around the world. The purpose of this study was to explore diet quality during the COVID-19 pandemic in 11 Latin American countries.

**Methodology:**

Multicentric, cross-sectional study. An online survey was applied to residents of 11 Latin-American countries, during April and May 2020, when confinement was mandatory. Diet quality was evaluated using a validated questionnaire.

**Result:**

10,573 people participated in the study. The quality of the food by country shows that Colombia presented the best quality, while Chile and Paraguay presented the lowest. When comparing the overall results of diet quality by gender, schooling and age, women, people with more schooling and people under 30 years of age, presented better diet quality. The regression model showed that the variables associated with diet quality were: age (*df* = 3, *F* = 4. 57, *p* < 0.001), sex (*df* = 1, *F* = 131.01, *p* < 0.001), level of education (*df* = 1, *F* = 38.29, *p* < 0.001), perception of weight change (*df* = 2, *F* = 135.31, *p* < 0.001), basis services (*df* = 1, *F* = 8.63, *p* = 0.003), and quarantine (*df* = 1, *F* = 12.14, *p* = 0.001).

**Conclusion:**

It is necessary for governments to intervene to reverse these indicators, considering that inadequate feeding favors the appearance of no communicable diseases, which favor a higher risk of infection and worse prognosis with COVID-19.

**Supplementary Information:**

The online version contains supplementary material available at 10.1186/s41043-022-00316-8.

## Background

Severe Acute Respiratory Syndrome Coronavirus 2 (SARS-CoV-2) was identified by Chinese authorities the 7th of January 2020 as the cause of new disease later knew as "coronavirus disease 2019" (COVID-19) [1]. The 30th of January 2020, the World Health Organization (WHO) declared the outbreak of COVID-19 a global health emergency [1]. As a result, countries implemented measures intended to contain the spread of the disease including, mobility restrictions, confinement, and cancellation of incoming flights from risk countries [2].

In Latin America the pandemic has been characterized by a large number of infections, high mortality and long periods of confinement [3, 4], which have affected food supply chain, food environments, as well as purchasing, selection, preparation and consumption of food [5]. Moreover, anxiety, frustration, fear and stress that occur quite often during emergency situations have induced unhealthy food choices and consumption, and conditioned eating behaviors such as binge eating and bulk buying episodes, among others [6].

Although Latin American countries are large producers of grains and fresh foods, consumption of unhealthy food including sugary drinks occurs quite often, clearly affecting quality of diet [7, 8]. In addition, the region has a high prevalence of obesity and chronic diseases [9], which can aggravate symptoms of infections [10]. As a result of not having a healthy and varied diet, which contributes to a weak immune system [11], it is likely that a large part of the population was not adequately protected against the disease. Such circumstance might contribute to a higher risk of contagion and bad prognosis in case of COVID-19 infection [12]. An optimal nutrition is important to help curbing the consequences of quarantines and preventing and control chronic diseases associated [13].

Although several studies have assessed the quality of the diet in different contexts, there is little information on this regard in the context of the COVID-19 pandemic specially in Latin American countries. The aim of this study was to explore diet quality among adults of 11 Latin American countries during the COVID-19 pandemic.

## Methodology

### Study design and setting

The present is a multi-centric, cross-sectional study, carried out during April and May 2020. Individuals were invited to participate through different platforms and social networks on a voluntary and anonymous basis. An online survey using Google Forms was used to collect data. The survey was self-administered and applied in a single opportunity between the 15th of April and 4th of May 2020.

### Study populations

Citizens residing in 11 countries (Argentina, Chile, Colombia, Costa Rica, Ecuador, Guatemala, Mexico, Peru, Paraguay, Panama, and Uruguay) were invited to participate in the study. All participants had to be over 18 years old and had to accept the informed consent to be able to participate. People who were pregnant and/or nursing, who were under pharmacological treatment or psychotherapies for depression, anxiety disorders, stress or mood disorders, and those with dietary treatment for pathologies were excluded from the study.

### Variables

#### Outcome

Main outcome variable was quality of diet. Quality of diet was evaluated using a closed food consumption frequency survey that was based on two previously validated questionnaires [14, 15]. The questionnaire included 13 multiple choice questions, from which eight were dedicated to evaluating healthy eating habits (consumption of recommended food groups), and five to evaluate unhealthy eating habits (consumption of not recommended food groups). To establish healthy thresholds of food consumption frequency (i.e., recommended frequency of consumption of a specific food group), the recommendations of current food guidelines of several Latin American countries were used. In the case that an intake recommendation for a specific food item was not available, the healthy threshold was established by the consensus of a group of experts [16].

Healthy eating habits were assessed measuring the frequency of consumption of recommended food groups (fruits, vegetables, dairy products, eggs, meat, legumes, breakfast and bread). Depending on the frequency of consumption a score was given to each of the questions in the following way: "no consumption" (1 point), "consumption less than recommended" (3 points), "suggested day/weekly portions" (5 points). In this way, the total score of the "healthy eating habits" subscale varied from 8 to 40 points (the higher the value, the healthier the eating habits).

Unhealthy eating habits were assessed measuring the frequency of consumption of foods or food groups identified as promoting chronic non-communicable diseases such as pizza, fried foods, alcohol, pastries, sugared drinks. Depending on the frequency of consumption a score was given to each of the questions in the following way: "consumption excess recommendation" (1 point), "consumption follow recommendation" (3 or 5 points depending on the quantity, with 5 give to no consumption). In this way, the total score of the "unhealthy eating habits" subscale varied from 5 to 25 points (the higher the value, the healthier the eating habits).

The final scores corresponded to the sum of "healthy eating habits" and "unhealthy eating habits" subscales. Healthy eating was rated from 44 to 65 points, moderate unhealthy from 23 to 43 points and unhealthy from 13 to 22 points.

#### Predictor variables

In the present study we analyzed the association of diet quality (outcome) and country of residence (main predictor). However, we also describe the association of diet quality with other predictors of interest including, age (years), sex (male, female), education level (basic/secondary, university), work (yes, no), basic services (some, all), confinement (yes, no), perception of weight change during the pandemic (did not change, increased, decreased).

#### Statistical analyses

To test differences between countries regarding socio-demographic variables we applied Chi2 test for categorical and Kruskall-Wallis for numeric variables. Results for these test are showed in Table 1. To evaluate the association between diet quality (outcome) and predictors (see Outcome and predictor variables) we fit a linear regression model, where diet quality was modeled as a continuous. Age was modeled as nonlinear variable including restrictive cubic splines. All analyses were performed using R and the package rms [17].

**Table 1:** General characteristics of the sample by country

	Argentina	Chile	Colombia	Costa Rica	Ecuador	Guatemala	Mexico	Panama	Paraguay	Peru	Uruguay	Test stat	*P* value
	1422	1033	573	576	577	963	1280	617	609	746	861		
**Sex**													
Female	1247 (87.69)	826 (79.96)	464 (80.98)	439 (76.22)	404 (70.02)	702 (72.9)	1046 (81.72)	439 (71.15)	492 (80.79)	561 (75.2)	737 (85.6)	Chisq. (10 *df*) = 179.704	< 0.001
Male	175 (12.31)	207 (20.04)	109 (19.02)	137 (23.78)	173 (29.98)	261 (27.1)	234 (18.28)	178 (28.85)	117 (19.21)	185 (24.8)	124 (14.4)		
**Age**													
Median(IQR)	38 (31,47)	31 (26,40)	31 (24,39)	43 (31,52)	29 (23,40)	31 (24,45)	31 (25,41)	28 (23,39)	34 (29,41)	36 (28,46)	41 (31,52)	Kruskal–Wallis test	< 0.001
**Education level**													
Basic/secondary	216 (15.19)	70 (6.78)	42 (7.33)	54 (9.38)	39 (6.76)	69 (7.17)	50 (3.91)	56 (9.08)	26 (4.27)	31 (4.16)	135 (15.68)	Chisq. (10 *df*) = 215.689	< 0.001
University	1206 (84.81)	963 (93.22)	531 (92.67)	522 (90.62)	538 (93.24)	894 (92.83)	1230 (96.09)	561 (90.92)	583 (95.73)	715 (95.84)	726 (84.32)		
**Work**													
No	381 (26.79)	384 (37.17)	204 (35.6)	140 (24.31)	230 (39.86)	216 (22.43)	455 (35.55)	301 (48.78)	189 (31.03)	256 (34.32)	217 (25.2)	Chisq. (10 *df*) = 213.622	< 0.001
Yes	1041 (73.21)	649 (62.83)	369 (64.4)	436 (75.69)	347 (60.14)	747 (77.57)	825 (64.45)	316 (51.22)	420 (68.97)	490 (65.68)	644 (74.8)		
**Basic services**													
Some	140 (9.85)	42 (4.07)	25 (4.36)	259 (44.97)	35 (6.07)	83 (8.62)	20 (1.56)	30 (4.86)	89 (14.61)	113 (15.15)	117 (13.59)	Chisq. (10 *df*) = 996.219	< 0.001
All	1282 (90.15)	991 (95.93)	548 (95.64)	317 (55.03)	542 (93.93)	880 (91.38)	1260 (98.44)	587 (95.14)	520 (85.39)	633 (84.85)	744 (86.41)		
**Confinement**													
Yes	1387 (97.54)	968 (93.71)	555 (96.86)	545 (94.62)	551 (95.49)	903 (93.77)	1199 (93.67)	590 (95.62)	587 (96.39)	720 (96.51)	773 (89.78)	Chisq. (10 *df*) = 87.879	< 0.001
No	35 (2.46)	65 (6.29)	18 (3.14)	31 (5.38)	26 (4.51)	60 (6.23)	81 (6.33)	27 (4.38)	22 (3.61)	26 (3.49)	88 (10.22)		
**Weight change**													
Did not changed	673 (47.33)	368 (35.62)	275 (47.99)	277 (48.09)	234 (40.55)	435 (45.17)	571 (44.61)	288 (46.68)	226 (37.11)	324 (43.43)	432 (50.17)	Chisq. (20 *df*) = 283.378	< 0.001
Increased	572 (40.23)	502 (48.6)	163 (28.45)	205 (35.59)	198 (34.32)	368 (38.21)	456 (35.62)	177 (28.69)	315 (51.72)	280 (37.53)	367 (42.62)		
Decreased	177 (12.45)	163 (15.78)	135 (23.56)	94 (16.32)	145 (25.13)	160 (16.61)	253 (19.77)	152 (24.64)	68 (11.17)	142 (19.03)	62 (7.2)		

For numerical variables, mean/median and standard deviation or interquartil range are showed (depending on the distribution of the variable). For categorical variables, frequency and percentages are showed

#### Ethical considerations

The study was developed following the Declaration of Helsinki, regarding work with human beings and according to the "Singapore Declaration on Integrity in Research." The study was approved by the Scientific Ethics Committee of the University of the Americas (Chile) and reviewed by local committees in the participating countries.

## Results

### General characteristics

Responses from 10,789 individuals were collected, from them 1532 were excluded because they belonged to individuals not living in the countries of interest. The final sample included 9257 individuals and completed the online questionnaire from the 11 invited countries. The majority of the sample was constituted by young, well-educated, females (Table 1). In addition, as can be observed in this table, most of the participants had a job and household accessibility to basic services and were in lockdown at the time of the survey. With regard to changes in body weight, participants from Paraguay showed the highest weight gain and those from Colombia showed the lowest weight gain.

Table 2 shows that Colombia has the highest score in diet quality and Paraguay obtained the lowest score. When observing the proportion between healthy and unhealthy diet (%) in Colombia, the percentage of participants with a healthy diet is three times greater than that of subjects with an unhealthy diet; by contrast, in Chile, Argentina, and Paraguay the proportion between healthy and unhealthy diet is similar.

**Table 2:** Diet quality by country

	Argentina	Chile	Colombia	Costa Rica	Ecuador	Guatemala	Mexico	Panama	Paraguay	Peru	Uruguay	Test stat	*P* value
	1422	1033	573	576	577	963	1280	617	609	746	861		
**Diet quality score**													
Median (IQR)	42 (39,46)	42 (39,46)	45 (42,48)	44 (41,47)	43 (40,46)	44 (41,47)	44 (40,47)	43 (40,46)	42 (38,45)	43.5 (41,46)	43 (39,47)	Kruskal–Wallis test	< 0.001
**Diet quality**													
Unhealthy	697 (49.02)	516 (49.95)	155 (27.05)	210 (36.46)	234 (40.55)	313 (32.5)	483 (37.73)	267 (43.27)	316 (51.89)	269 (36.06)	383 (44.48)	Chisq. (10 df) = 198.687	< 0.001
Healthy	725 (50.98)	517 (50.05)	418 (72.95)	366 (63.54)	343 (59.45)	650 (67.5)	797 (62.27)	350 (56.73)	293 (48.11)	477 (63.94)	478 (55.52)		

For numerical variables, mean/median and standard deviation or interquartil range are showed (depending on the distribution of the variable). For categorical variables, frequency and percentages are showed

**Fig. 1:**
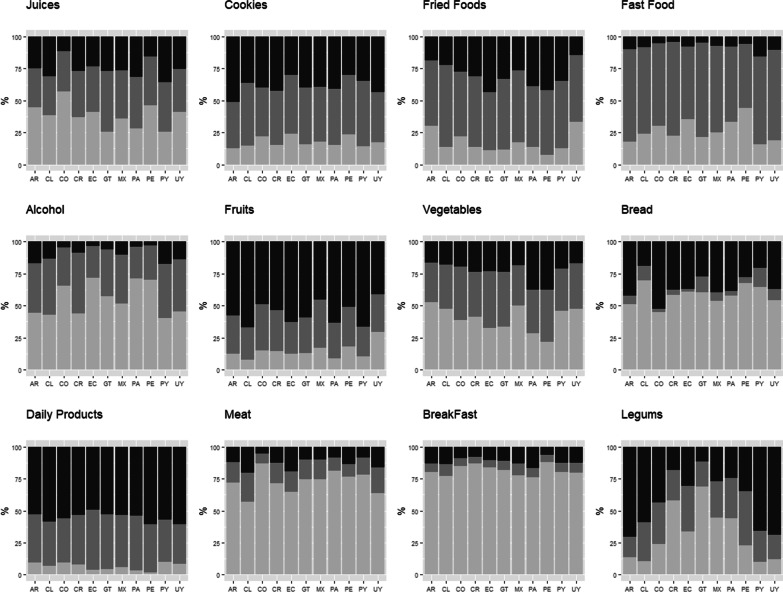
Comparison of the frequency of food consumption in selected countries. Note 1: Juices, Cookies, fried food, Fast food, Alcohol: "no consumption" (gray), "occasional consumption" (dark gray) y “frequently consumes” (black); Note 2: Fruits, Vegetables, bread, dairy, Meat, Breakfast, Legumes "no consumption" (black), "consumption less than recommended" (dark gray), to "suggested day/weekly portions" (gray). Abbreviations: AR: Argentina; CL: Chile; Co: Colombia; CR: Costa Rica; EC: Ecuador; GT: Guatemala; MX: Mexico; PA: Panama; PE: Peru; PY: Paraguay; UY: Uruguay; F: female M: male

Breakfast habit was common among respondents of Peru, Costa Rica, Colombia, and Ecuador (Fig. 1), while sporadic or no alcohol intake occurred mostly among respondents of Ecuador, Panama, and Peru (Fig. 1). Insufficient consumption of dairy products was mainly observed among participants from Uruguay and Peru (Fig. 1). Recommended fruit consumption predominated in Uruguay and legume consumption in Guatemala and Costa Rica (Fig. 1). In relation to the consumption of unhealthy foods, consumption of cookies and fried foods occurred quite often in Uruguay, Argentina, Peru, and Ecuador. Consumption of fried foods was high in Ecuador, Peru, Panama, and Paraguay (Fig. 1).

### Association between diet quality, country, and socio-demographic characteristics

**Fig. 2:**
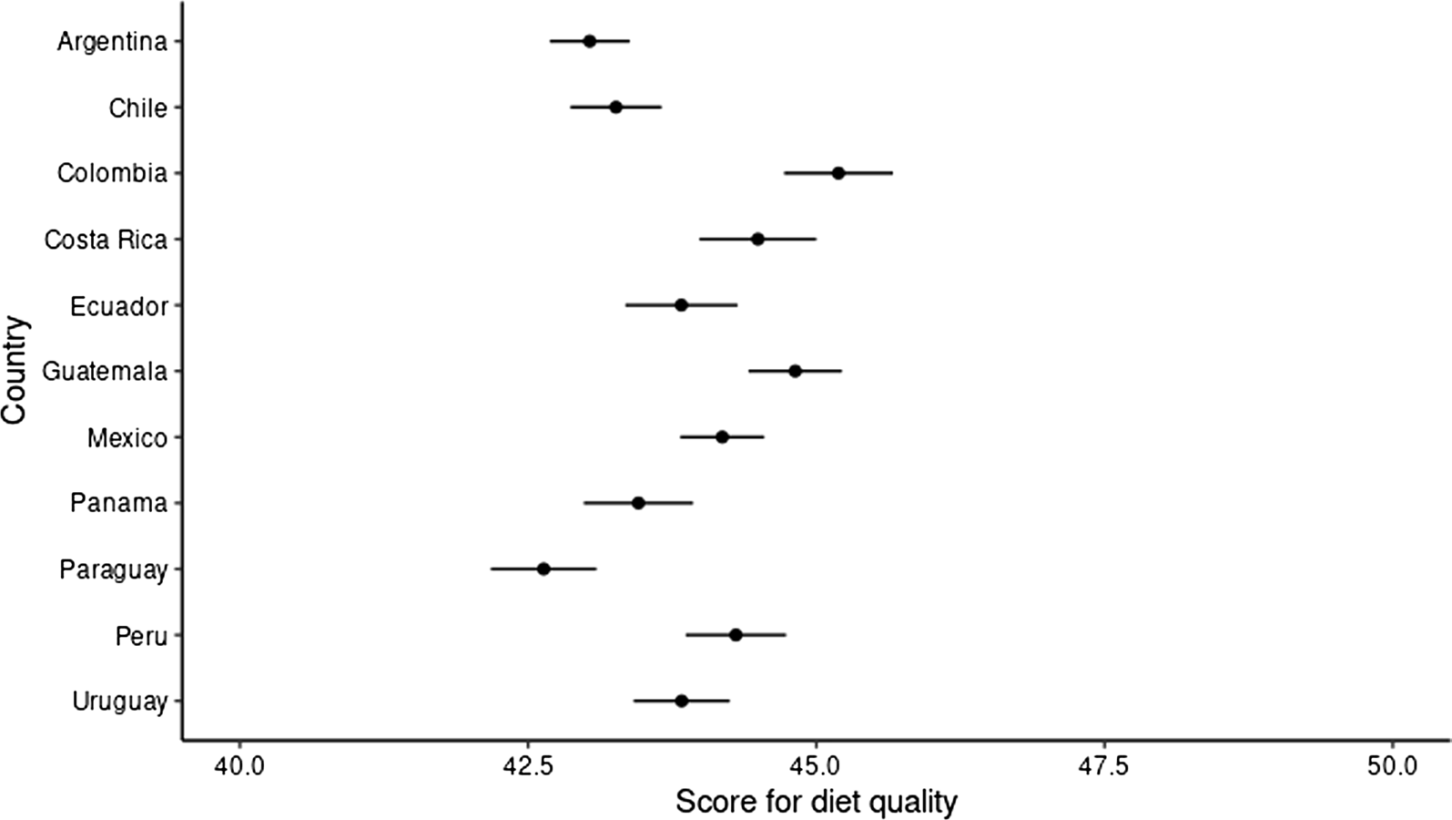
Adjusted mean scores of diet quality by country. Horizontal lines represent 95% confidence intervals. The highest the score the better the quality of the diet

Independently from socio-demographic confounders (see Methods), diet quality was significantly associated with country (*df* = 10, *F* = 17. 88, *p* < 0.001). From all Latin American countries, Colombia showed the highest diet quality score, suggesting that Colombians had the healthiest diet among all participants. As individuals from Colombia showed the healthiest diet, we set up Colombia as reference to understand differences among countries. We found all the countries, except to Guatemala, showed a less healthy quality of diet than Colombia as they had lower adjusted scores than Colombia (Fig. 2).

Diet quality was also significantly associated to age (*df* = 3, *F* = 4. 57, *p* < 0.001), sex (*df* = 1, *F* = 131.01, *p* < 0.001), level of education (*df* = 1, *F* = 38.29, *p* < 0.001), perception of weight change (*df* = 2, *F* = 135.31, *p* < 0.001), basis services (*df* = 1, *F* = 8.63, *p* = 0.003), and quarantine (*df* = 1, *F* = 12.14, *p* = 0.001). In this way, individuals younger than 30 years and older than 60 years showed healthier quality of diet than individuals between 30 and 60 years old (Additional file 1: Table S1). In comparison with males, and to individuals with lower level of education, females, and individuals with higher level of education showed a healthier quality of diet (Additional file 1: Table S1).

While individuals who reported having gain weight showed less healthy diet quality in comparison with people who reported no weight change, diet quality was similar among those who reported having loss weight or those who reported no weight change. Individuals who reported having all basic services showed healthier diet quality than those who reported having some of the basic services. Individuals who reported being confined showed healthier diet quality in comparison with those that reported not being confined (Additional file 1: Table S1). We did not find evidence for an association between diet quality and working status (*df* = 1, *F* = 0.05, *p* = 0.817).

## Discussion

During the COVID-19 pandemic, Latin-American countries have a poor quality diet, especially in men and in participants with lower levels of education. These results are consistent with previous observational studies on dietary quality [18–20]. Colombia is the country with the highest dietary quality and, in contrast, Chile and Paraguay have the lowest values. In addition, there is a low consumption of fruits and dairy products in all countries and a high consumption of fast-food juices and alcohol.

The results obtained in our survey, could be influenced by eating under a state of anxiety or boredom derived from long periods of confinement, by a decrease in motivation to maintain a healthy diet or by an increase in diet driven by mood [6].

Colombia is noted as the country with the best diet quality during this period; other studies have assessed the behavior of Colombians during the pandemic, and their findings showed an increase in the consumption of fruits and a decrease in the consumption of vegetables in adolescents [21], and in the case of adults there was an increase in healthy food such as pulses, cereals, and eggs; however, the consumption of fish and nuts decreased [22]. On the contrary, Paraguay showed the worst diet quality; in this country there is no much literature about dietary habits since a national food consumption survey has not been conducted yet. Some papers about specific age groups and a study carried out with medical doctors show that a great proportion of the population had inappropriate consumption patterns [23], and that Paraguay was the leading consumer of sugary drinks in the region during the pandemic [24]. Chile is the leader in consumption of sugary drinks, bread, and other unhealthy foods, despite being one of the pioneering countries in implementing mandatory front-of-package labeling [25].

Individuals and families who may not have previously experienced food insecurity now face limited windows to buy groceries, as well as empty shelves and long lines of panicked shoppers storing their "quarantine pantry." Previous research on food security suggests that this contributes to a pattern of "feast or famine," with alternating periods of food abundance, hoarding, and overconsumption with compensatory behaviors, followed by food shortages, skipping meals, and dietary restrictions [26].

Although authorities in several countries informed the population that there was no risk of food supply, studies have shown that this pandemic has had an effect on food systems, reducing the supply of fresh food [27], generating widespread anxiety and unhealthy eating behaviors such as an increase in highly palatable foods and alcohol [28, 29].

An Italian study showed that, during COVID-19 quarantines, the population reported an increase in the consumption of snacks and sweets [19]. Foods that occur in high frequency in our respondents.

The article by Coelho-Ravagnani and colleagues indicates that various nutrition societies encourage fruit and vegetable consumption [30]. However, our results show a low consumption of fruit in particular and a low percentage of individuals consuming at least 5 portions of fruit and vegetables. Fruits and vegetables are good sources of water, antioxidants and fiber, which play a role in immunity and the control of hypertension, diabetes, weight gain, some of the most important risk factors for complications of COVID-19 [31]. This low consumption may be due to the fact that it was already insufficient before the pandemic and is only the maintenance of this eating habit or is the result of the reduction in the supply of fruits and vegetables due to breaks in the production and distribution chain, the closure of supermarkets, markets or free trade fairs [27]. Another cause could also be the rise in the price of these foods, which mainly affects people with lower incomes or educational levels [32].

Adequate food can help maintain good immunity to viruses [11], however, price increases, food shortages, and poor dietary practices in the population may not be factors favoring good immunity. Adequate food, both in quantity and quality, is a fundamental pillar of maintaining good health. Inadequate food intake, among other aspects, reduces immunity and increases vulnerability to both communicable and non-communicable diseases [31]. It has been shown that specific nutrients or combinations of nutrients can affect the immune system through cell activation, modification in the production of signaling molecules and gene expression [33].

Recent studies show an increased risk of COVID-19 infection and a worse prognosis in people with type 2 diabetes mellitus, high blood pressure, obesity and pathologies with compromised immune systems. Obesity specifically has been associated with worse immune response and poor prognosis for respiratory infections [10, 34].

### Strengths and limitations of the study

Among the strengths of the study is the use of a previously validated document that is easy to understand and can be used for online surveys. In addition, our study was conducted during the most critical period of the pandemic. Among the weaknesses we can mention that online studies exclude people with less access to connectivity (less education or income) and older adults; furthermore, it is important to mention that the proportion of responses from women to men is higher, which did not allow for further analysis.

## Conclusions

In general, there is a poor quality diet. Furthermore, men, individuals with less schooling and those who have increased their weight or changed their diet are those who have a lower quality diet, specifically a low consumption of fruits, vegetables and dairy products and a high consumption of pastries and fried foods.

Countries need to take action to reverse these indicators, both by increasing nutrition education, incorporating subsidies, and ensuring healthy food supplies.

## Supplementary Information

**Additional file 1**. **Supplementary table 1.** Adjusted predicted means of diet quality score.

## Data Availability

The datasets used during the current study are available from the corresponding author on reasonable request.

## Abbreviations

SARS-CoV-2: Severe Acute Respiratory Syndrome Coronavirus 2
COVID-19: Coronavirus disease 2019
WHO: World Health Organization
AR: Argentina
CL: Chile
Co: Colombia
CR: Costa Rica
EC: Ecuador
GT: Guatemala
MX: México
PA: Panamá
PE: Perú
PY: Paraguay
UY: Uruguay
F: Female
M: Male
