# Supplementary material for: Quality of the diet during the COVID-19 pandemic in 11 Latin-American countries
Source: J Health Popul Nutr. 2022 Aug 4;41:33. doi: 10.1186/s41043-022-00316-8 (PMC9351126; doi:10.1186/s41043-022-00316-8)
Supplement: Supplementary file 1 — Additional file 1. Supplementary table 1. Adjusted predicted means of diet quality score. [file 41043_2022_316_MOESM1_ESM.docx]

**Supplementary table 1. Adjusted predicted means of diet quality score.** Abbreviations: LCI, lower 95% confidence interval; UCI, upper 95% confidence interval

|  |  | **Mean** | **LCI** | **UCI** |
| --- | --- | --- | --- | --- |
| Age | 20 | 45,61 | 45,08 | 46,15 |
|  | 30 | 45,09 | 44,63 | 45,55 |
|  | 40 | 45,32 | 44,86 | 45,79 |
|  | 50 | 45,57 | 45,09 | 46,06 |
|  | 60 | 45,93 | 45,43 | 46,43 |
|  | 70 | 46,31 | 45,64 | 46,98 |
|  | 80 | 46,69 | 45,77 | 47,61 |
|  | 90 | 47,07 | 45,86 | 48,28 |
| Sex | Female | 45,19 | 44,72 | 45,66 |
|  | Male | 43,69 | 43,18 | 44,20 |
| Education level | Basic/secondary | 44,00 | 43,40 | 44,59 |
|  | University | 45,19 | 44,72 | 45,66 |
| Work | No | 45,22 | 44,72 | 45,72 |
|  | Yes | 45,19 | 44,72 | 45,66 |
| Basic services | Some | 44,66 | 44,07 | 45,24 |
|  | All | 45,19 | 44,72 | 45,66 |
| Weight change | Did not change | 45,19 | 44,72 | 45,66 |
|  | Increased | 43,57 | 43,09 | 44,05 |
|  | Decreased | 45,65 | 45,15 | 46,16 |
| Confinement | Yes | 45,19 | 44,72 | 45,66 |
|  | No | 44,36 | 43,71 | 45,00 |
